# Microbiomes in action: multifaceted benefits and challenges across academic disciplines

**DOI:** 10.3389/fmicb.2025.1550749

**Published:** 2025-03-18

**Authors:** Sereyboth Soth, John G. Hampton, Hossein Alizadeh, Steve A. Wakelin, Artemio Mendoza-Mendoza

**Affiliations:** ^1^Faculty of Agriculture and Life Sciences, Lincoln University, Lincoln, Canterbury, New Zealand; ^2^National Institute of Science, Technology and Innovation, Phnom Penh, Cambodia; ^3^Scion, Christchurch, New Zealand

**Keywords:** agriculture, biocatalyst processes, environmental bioremediation, food processing, human health, microbes, microbiomes

## Abstract

Microbiomes combine the species and activities of all microorganisms living together in a specific habitat. They comprise unique ecological niches with influences that scale from local to global ecosystems. Understanding the connectivity of microbiomes across academic disciplines is important to help mitigate global climate change, reduce food insecurity, control harmful diseases, and ensure environmental sustainability. However, most publications refer to individual microbiomes, and those integrating two or more related disciplines are rare. This review examines the multifaceted benefits of microbiomes across agriculture, food manufacturing and preservation, the natural environment, human health, and biocatalyst processes. Plant microbiomes, by improving plant nutrient cycling and increasing plant abiotic and biotic stress resilience, have increased crop yields by over 20%. Food microbiomes generate approximately USD 30 billion to the global economy through the fermented food industry alone. Environmental microbiomes help detoxify pollutants, absorb more than 90% of heavy metals, and facilitate carbon sequestration. For human microbiomes, an adult person can carry up to 38 trillion microbes which regulate well being, immune functionality, reproductive function, and disease prevention. Microbiomes are used to optimize biocatalyst processes which produce bioenergy and biochemicals; bioethanol production alone is valued at over USD 83 billion p.a. However, challenges, including knowledge gaps, engaging indigenous communities, technical limitations, regulatory considerations, the need for interdisciplinary collaboration, and ethical issues, must be overcome before the potential for microbiomes can be more effectively realized.

## Graphical Abstract

Multifaceted benefits of microbiomes across academic disciplines for a sustainable world. Unlocking the full potential of microbiomes will contribute to achieving sustainable development goals such as zero hunger, good health and well being, affordable and clean energy, and mitigation of climate change. 
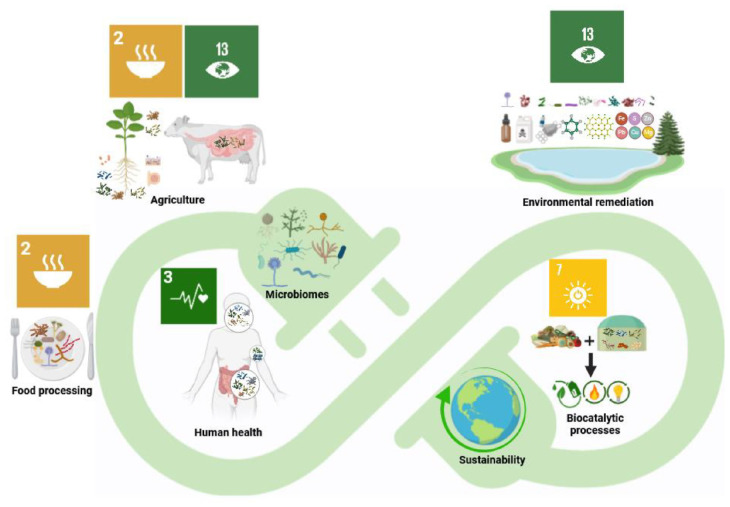

## 1 Introduction

A microbiome is the combination of all the microorganisms living together in a particular habitat. The expression of their collective activities and interactions creates unique ecological niches that impact surrounding ecosystems (Berg et al., 2020). All plants and animals establish symbiotic associations with an extensive diversity of microorganisms, forming functional integrated super-organismal units termed holobionts (the combination of an eukaryotic organism and its microbiome comprising a single ecological unit) (Berg et al., 2017; Poole, 2017; Zilber-Rosenberg and Rosenberg, 2008; Baedke et al., 2020; Gilbert, 2014; Brinker et al., 2019). The concept of symbiosis is now considered the rule rather than the exception and is emerging as one of the central principles of contemporary biology (Gilbert, 2014).

In both natural and productive (e.g., agricultural) environments, plant microbiomes improve plant nutrient acquisition and protect plants against biotic and abiotic stresses. They enhance growth, productivity, and biodiversity, delivering a wide range of ecosystem functions (Salas-Gonzalez et al., 2021; Mendoza-Mendoza et al., 2024; Chieb and Gachomo, 2023; Gibert et al., 2019). Microbiomes are key players in many aspects of food manufacturing and preservation, contributing to the development of various fermented foods and extending the shelf life of many food products. People consume food shaped by microbiomes daily (Siddiqui et al., 2023), deriving value from distinct flavor profiles, prolonged preservation, and nutritional advantages conferred by microorganisms in the context of fermented foods (e.g., cheese, pickled vegetables and fruit, tempeh, and yogurt) and beverages (e.g., beer, cider, whiskey, and wine) (Louw et al., 2023; Piao et al., 2015; Pinto et al., 2015). Humans are profoundly influenced by their microbiomes. An adult human weighing around 70 kilograms may have up to 38 trillion microbes from 3,000 species (Sender et al., 2016), all influencing health and wellbeing. For example, the gut, skin, oral, and vaginal microbiomes influence digestion, nutrient absorption, immune system regulation, and reproductive functions (Zheng et al., 2020; Coelho et al., 2021; Ma et al., 2023). Current findings suggest that the gut microbiota influence mood and mental health, including anxiety, depression, bipolar disorder, schizophrenia, and obsessive-compulsive disorder, among others (Gupt et al., 2024). Microbiomes help create toxic-free ecosystems, such as in bioremediation for environmental cleanup and wastewater treatment (Rani et al., 2019; Ayilara and Babalola, 2023; Abatenh et al., 2017; Xu et al., 2014; Hu et al., 2021; Cheng et al., 2022; Ruan et al., 2024). A summary of key microbiome taxa and their roles across academic disciplines is presented in Table 1.

**TABLE 1:** A summary of microbiome taxa and their roles across academic disciplines.

Academic discipline	Microbial taxa	Microbial role	References
**Agriculture**			
**Nutrient regulation**			
	*Bradyrhizobium* spp.	Nitrogen fixation	Andrews and Andrews, 2017
	*Chlamydomonas reinhardtii*	Phosphate starvation response	Paries and Gutjahr, 2023
	*Azospirillum* spp*., Azotobacter* spp*., Bacillus* spp*., Paenibacillus* spp*., Acidithiobacillus ferrooxidans, Pseudomonas* spp., and *Burkholderia* spp.	Potassium, phosphorous, and zinc solubilization	Meena et al., 2016
	*Bacillus amyloliquefaciens*	Sulfur assimilation	Aziz et al., 2016
	*Piriformospora indica*	Magnesium transportation	Prasad et al., 2018
	*Pseudomonas fluorescens, Rhizobium leguminosarum*, and *Azospirillum lipopherum*	Copper and iron translocation	Carrillo-Castañeda et al., 2002
	*Glomus* spp.	Manganese uptake	Nogueira and Cardoso, 2003
	*Pseudomonas fragi, Pantoea dispersa, Pantoea agglomerans, Enterobacter cloacae*, and *Rhizobium* spp.	Zinc solubilization	Kamran et al., 2017
	*Claroideoglomus etunicatum*	Molybdenum uptake	Shi et al., 2020
	*Rhizophagus irregularis*	Boron transportation and homeostasis	Quiroga et al., 2020
	*Psychrobacter* spp. and *Bacillus cereus*	Nickel mobilization	Ma et al., 2009
**Pathogen and invertebrate pest control**			
	*Trichoderma* spp.	Control an array of plant pathogens	Mendoza-Mendoza et al., 2024; Moran-Diez et al., 2021
	Combination of rhizosphere microbiome	Control *Ralstonia solanacearum*	Jiang et al., 2022
	*Bacillus* spp. and *Pseudomonas* spp.	Inhibit growth of *Fusarium* spp. and *Pythium* spp.	Chen et al., 2024
	*Bacillus* spp*., Beauveria* spp., and *Metarhizium* spp.	Control an array of insect pests	Santoyo, 2022; Chen et al., 2023; Shi et al., 2021
	*Fusarium oxysporum*	Control *Pratylenchus goodeyi* and *Helicotylenchus multicinctus*	Waweru et al., 2014
	*Epichloë* spp.	Control various grass root feeding pests	Johnson et al., 2013
**Enhancing plant resilience to abiotic stresses**			
	Plant growth-promoting rhizobacteria	Enhance plant drought stress resilience	Chieb and Gachomo, 2023
	Forest and agricultural microbiomes	Enhance plant drought stress resilience	Carter et al., 2023
	*Bacillus* spp., *Mycobacterium* spp., *Paenibacillus* spp., *Alcaligenes* spp., *Acidovorax* spp., *Rhodococcus* spp., and *Pseudomonas* spp.	Detoxification of phytotoxins	Sharma et al., 2021
	*Pseudomonas fluorescens* and *Bacillus subtilis*	Improve plant salinity stress resilience	Saberi-Riseh et al., 2020
	*Pseudomonas fluorescens*	Improve plant tolerance to heat stress	Chen J. et al., 2019
**Regulating phytohormones**			
	*Variovorax* spp., and *Pseudomonas* spp.	Auxin regulation	Gonin et al., 2023
	*Azospirillum* spp., *Bacillus* spp., and *Pseudomonas* spp.	Cytokinin regulation	Gupta et al., 2022; Hussain and Hasnain, 2011
	*Bacillus* spp., *Azospirillum* spp*., Aspergillus fumigatus, Penicillium* spp*., Pseudomonas* spp*., and Rhizobium* spp.	Gibberellin regulation	Keswani et al., 2022; Cohen et al., 2009; Khan et al., 2011; Kapoor et al., 2016; Yanni et al., 2001
	*Rhodococcus* spp. and *Novosphingobium* spp.	Abscisic acid regulation	Belimov et al., 2014
	*Streptomyces* spp., *Bacillus* spp., and *Lysinibacillus* spp.	Jasmonic acid interaction	Carvalhais et al., 2015
	*Rhodanobacter* spp., *Sphingomonas* spp., and *Micromonospora* spp.	Salicylic acid interaction	Zhu et al., 2022
	*Azospirillum* spp., *Rhizobium* spp., *Agrobacterium* spp., *Achromobacter* spp., *Burkholderia* spp., *Ralstonia* spp., *Pseudomonas* spp., *Enterobacter* spp., and *Kluyvera ascorbata*	Ethylene regulating encoding genes	Blaha et al., 2006
**Food production**			
	*Aspergillus* spp., and *Bacillus* spp.	Soy sauce production	Yee et al., 2021
	*Enterococci* spp. and *Rhizopus* spp.	Tempeh production	Moreno et al., 2002; Nout and Aidoo, 2011
	*Lactobacillus* spp.	Fruit and vegetable pickle	Giraffa et al., 2010
	*Lactobacillus spp.* and *Penicilium* spp.	Cheese production	Ropars et al., 2020; Bautista-Gallego et al., 2014
	*Lactobacillus* spp. and *Streptococcus* spp.	Yogurt production	Mena and Aryana, 2012
	*Lactobacillus* spp. and *Saccharomyces* spp.	Sourdough bread production	Aplevicz et al., 2013
**Beverage production**			
	*Saccharomyces* spp. and *Oenococcus* spp.	Wine production	du Toit et al., 2011; Fleet, 2008
	*Lactobacillus* spp. and *Saccharomyces* spp.	Whiskey production	Liu et al., 2023; Walker and Hill, 2016
	*Lactobacillus* spp. and *Saccharomyces* spp.	Beer and cider production	Suiker and Wösten, 2022
**Future food microbes**			
	*Spirulina* spp., *Chlorella* spp., and *Scenedesmus* spp.	Source of fiber and micronutrients	Ravindra, 2000; Graham and Ledesma-Amaro, 2023
	*Cellulomonas* spp. and *Alcaligenes* spp.	Source of protein and micronutrients	Ravindra, 2000; Graham and Ledesma-Amaro, 2023
	*Aspergillus* spp., *Penicillium* spp., and *Trichoderma* spp.	Source of fiber, protein, and micronutrients	Ravindra, 2000; Graham and Ledesma-Amaro, 2023
	*Saccharomyces* spp. and *Pichia* spp.	Source of fiber, protein, and micronutrients	Ravindra, 2000; Graham and Ledesma-Amaro, 2023
**Environment**			
**Detoxification of toxins**			
	*Rhodococcus rhodochrous* and *Aspergillus fumigatus*	Detoxifying heavy metals and metalloids	Hartmann and Six, 2023
	*Bacillus* spp., *Enterobacter* spp*., Klebsiella* spp*., Pseudomonas* spp. and *Phyllobacterium myrsinacearum*	Detoxifying of phytotoxins	Sarathambal et al., 2017; Jing et al., 2014; Manzoor et al., 2019; Ma et al., 2013
	*Enterobacter* spp., *Azospirillum* spp., *Pseudomonas* spp., *Klebsiella* spp., and *Vibrio* spp.	Cleansing wastewater	Haque et al., 2020; Lamers et al., 2012
**Pollutant degradation**			
	*Ideonella sakaiensis*	Breaking down plastic waste	Yoshida et al., 2016
	*Bacillus* spp., *Mycobacterium* spp., *Pseudomonas* spp., and *Nocardia* spp.	Degrading pesticides in water and soil	Bala et al., 2022
	*Aspergillus tubingensis, Chlorella vulgaris*, and *Lysinibacillus* spp.	Removing heavy metals	Mahanty et al., 2020; Govarthanan et al., 2020; San Keskin et al., 2018
**Human health**			
**Gut microbiomes and human health**			
	*Faecalibacterium* spp.	Prevent inflammatory bowel disease	Martín et al., 2023
	*Ruminococcus* spp.	Metabolize carbohydrate and fiber	Valentino et al., 2024
	*Clostridium* spp.	Minimize inflammation and allergic diseases	Guo et al., 2020
	*Eubacterium* spp.	Produce butyrate to balance energy use, colonic activities, immune function, and prevent gut inflammation	Mukherjee et al., 2020
	*Bacteroides* spp.	Regulate immunity, metabolize glucose and lipid	Wang et al., 2021
	*Prevotella copri*	Glucose homeostasis and host metabolism	Asnicar et al., 2021
	*Bifidobacterium* spp.	Digest fiber and support immune function	O’Callaghan and Van Sinderen, 2016
	*Akkermansia muciniphila*	Improves intestinal mucus layer	Karcher et al., 2021
**Microbiomes in therapeutics**			
	*Bacillus subtilis* and *Streptococcus faecium*	Reduce abdominal pain	Choi et al., 2015
	*Lactobacillus plantarum*	Decreases sepsis in newborn babies	Panigrahi et al., 2017
**Skin, oral, and vaginal microbiomes**			
	*Propionibacterium acnes*, *Staphylococcus epidermidis*, and *Staphylococcus aureus*	Influence immune responses and skin conditions	Byrd et al., 2018; Smythe and Wilkinson, 2023
	*Streptococcus* spp.	Assemble oral microbiomes	Abranches et al., 2018
	*Veillonella* spp.	Utilize lactic acid to protect teeth health	Giacomini et al., 2023
	*Lactobacillus* spp. and *Gardnerella vaginalis*	Impact reproductive health	Chen et al., 2021
**Biocatalyst processes**			
**Bioenergy production**			
	*Firmicutes* and *Bacteroides*	Hydrolyze complex organic materials	Mamindlapelli et al., 2021
	*Chloroflexi* spp., *Proteobacteria* spp., and *Atribacteria* spp.	Synthesize broken down organic substances into volatile fatty acids	Lim et al., 2020
	*Acetobacterium* spp., *Clostridium* spp., and *Syntrophomonas* spp.	Catabolize volatile fatty acids	Lim et al., 2020
	*Methanobacterium* spp., *Methanosarcina* spp., and *Methanococcus* spp.	Transform acetate, carbon dioxide, and hydrogen into bioenergy	Lim et al., 2020
**Bioethanol production**			
	*Trichoderma reesei*	Produces an enzyme that breaks down lignocellulosic biomass	Rastogi and Shrivastava, 2017
	*Saccharomyces cerevisiae*	Utilizes hexose sugar	Rastogi and Shrivastava, 2017
	*Scheffersomyces stipites*	Exploits pentose sugar	Rastogi and Shrivastava, 2017
	*Penicillium echinulatum*	Produces cellulase and xylanase	Todero Ritter et al., 2013
	*Anoxybacillus flavithermus*	Stabilizes pH and temperature for xylanase synthesis	Ellis and Magnuson, 2012
	*Aspergillus* spp. and *Kluyveromyces* spp.	Synthesize inulin	Ricca et al., 2007
**Biomaterial production**			
	*Cupriavidus necator, Pseudomonas putida, Aeromonas hydrophila and Pseudomonas aeruginosa*	Biosynthesize polyhydroxyalkanoates into bioplastic	Philip et al., 2007; Manoli et al., 2020; Chen, 2010
	*Bacillus* sp., *Aneurinibacillus* spp., and *Trichoderma harzianum*	Produce lignin-degrading enzymes	Sharma et al., 2022

Soil and environmental microbiomes improve soil health, degrade phytotoxicity, and enhance plant nutrient availability (Table 1). These collectively contribute to sustainable food production. This toxin-free environment nurtures plant microbiomes that support plant growth, builds stress resilience, and increases plant yield. The plant microbiome transforms it into highly nutritious and healthy diets for humans and animals. When humans consume plants, they consume plant holobionts, impacting their gut microbiomes and functioning, playing a key role in maintaining well being and immunity. Waste generated by this process and other agricultural byproducts is transformed into bioenergy or biofuel by biocatalyst microbiomes. Finally, the final waste turns into biofertilizer, which increases plant productivity and improves microbiome diversity in the environment and soil. This interconnected process is indefinitely cyclical. Recent One Health research has recognized the important of this interconnected nature of microbiomes for soil, plants, animal, and human health (van Bruggen et al., 2019; Law et al., 2024; Ma et al., 2023). However, these studies tended to emphasize the role of microbiomes for health-related outcomes rather than explicit conceptualization of their cyclical nature in sustaining ecosystem functions. Therefore, this article introduces the “circular microbiome system” concept, which refers to the interconnectedness of microbiomes across life science disciplines in a virtuous and interdependent cycle. For example, the inclusion of biocatalyst microbiomes that transform waste into bioenergy or biofertilizer and the use of indigenous knowledge to complete the loop. Despite this inherent interconnection, microbiome systems are often studied in isolation. To fully leverage the potential of microbiomes, we need a holistic approach that formally integrates interconnected domains, recognizing the microbiome as a unified, interconnected system that sustains life on Earth. As microbiomes play a fundamental role in optimizing sustainability, the understanding and utilization of microbiomes offer tremendous opportunities for improving food production, environmental remediation, human health, and biocatalyst processes. This review examines the multifaceted application of microbiomes involved in the sustainability of diverse ecosystems and identifies challenges that may delay the potential use of microbiomes.

## 2 Microbiomes in agriculture and food production

**FIGURE 1:**
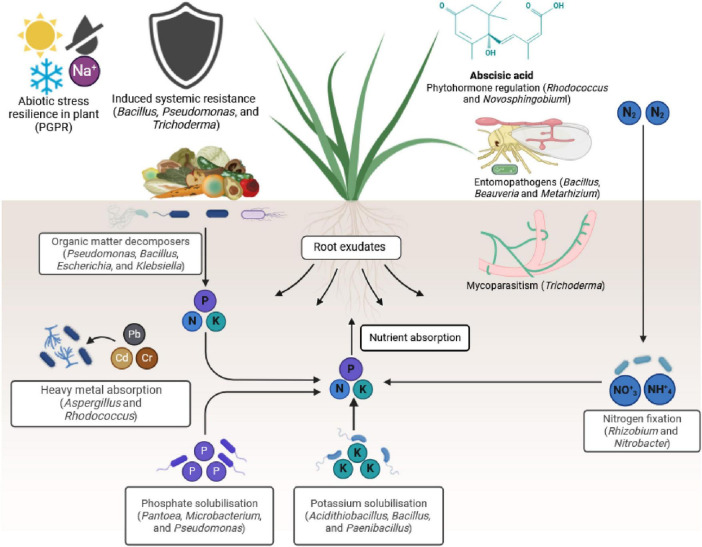
Mechanisms by which plant microbiomes can improve plant health for sustainable agricultural systems. They: (1) play a role in nutrient cycling, (2) are involved in pest and disease suppression, (3) induce plant resistance to abiotic stresses, (4) regulate phytohormones to respond to stresses, and (5) form beneficial biofilms (Figure was created using Biorender at https://app.biorender.com/).

**FIGURE 2:**
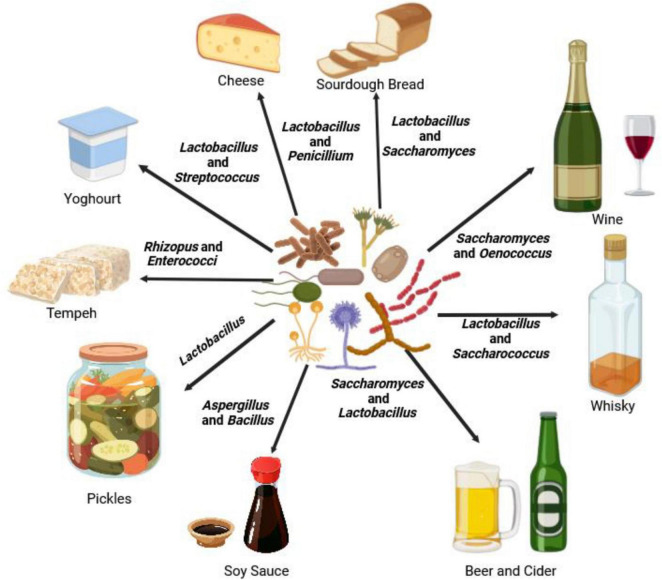
An illustration of how microbiomes such as *Aspergillus, Bacillus* (Yee et al., 2021), *Enterococci* (Moreno et al., 2002), *Lactobacillus* (Giraffa et al., 2010), *Oenococcus* (du Toit et al., 2011), *Penicillium* (Ropars et al., 2020), *Rhizopus* (Nout and Aidoo, 2011), *Saccharomyces* (Walker and Stewart, 2016), and *Streptococcus* (Mena and Aryana, 2012) are used for food (bread, cheese, pickles, soy sauce, tempeh, and yogurt) and beverages (beer, cider, wine, and whiskey) processing.

Microbiomes play a significant role in enhancing nutrient cycling, suppressing diseases and insect pests, promoting stress tolerance, regulating phytohormones (Figure 1), and facilitating food processing (Sessitsch et al., 2023; Ayilara and Babalola, 2023; Munir et al., 2022; Kulkarni et al., 2024; Bhat et al., 2022; Solomon et al., 2023; Molefe et al., 2023; Suman et al., 2022; Figure 2). For instance, plant microbiomes help increase staple crop yield by up to 20% under field conditions (Trivedi et al., 2017).

### 2.1 Nutrient cycling

Microbiomes are integral to ecosystem nutrient transport, mineralization, and solubilization. For example, mycorrhizal fungi exchange resources with plants by facilitating the solubility and transport of insoluble soil nutrients (Aislabie and Deslippe, 2013), particularly phosphorus, to the plant root system. The mycorrhizal networks associated with roots can increase the effective volume of soil the root can access by orders of magnitude (Li et al., 1991). Similarly, the interaction of nutrients in the soil, such as nitrogen, phosphorus, or sulfur, dramatically alters their solubility and chemical reactivity, directly influencing their transport in the environment and/or movement and exchange between the biosphere, atmosphere, soil, and water.

Microbiomes support nutrient cycling by breaking down and transforming complex organic compounds into forms that plants can utilize as they have a high-to-low carbon-to-nitrogen (C:N) ratio of 12:1 or 8:1 and a short shelf life (Raza et al., 2023). Additionally, microbiomes can transform insoluble nutrients into plant-available soluble ones. Plant root bacterial families such as Xanthobacteraceae and Bryobacteraceae have genes related to organic compound intake and phosphorus, and nitrogen turnover, which function as phosphorus transporters, mineralizers, and solubilizers (Camargo et al., 2023). Besides enhancing the availability of macronutrients like nitrogen (Andrews and Andrews, 2017), phosphorus (Paries and Gutjahr, 2023; Finkel et al., 2019), potassium (Masood and Bano, 2016), sulfur (Aziz et al., 2016), and magnesium (Prasad et al., 2018), plant microbiomes also improve the availability of micronutrients such as iron (Rajkumar et al., 2010), copper (Ferrol et al., 2016), manganese (Nogueira and Cardoso, 2003), zinc (Kamran et al., 2017), molybdenum (Shi et al., 2020), boron (Quiroga et al., 2020), and nickel (Singh et al., 2022). For example, inoculation of plants with strains of *Pseudomonas, Arthrobacter*, and *Bacillus* spp. increased nitrogen, phosphorus, and potassium content in *Astragalus mongholicus* seedlings by 8% to 118% (Shi et al., 2023). Under field conditions in a tropical savanna climate, inoculating soybeans with N-fixation bacteria increased yield by 22% (439 kg per hectare) over that of uninoculated plants (Cordeiro and Echer, 2019). In an analysis of 97 peer-reviewed papers about the use of microbes to improve crop yield, Li et al. (2022) reported that around 22% of them focused on microbes which improved plant nutrient availability, for which key players were *Enterobacter* (around 27%) and *Bacillus* (around 26%). Therefore, plant microbiomes contribute substantially to improving plant nutrient availability and could contribute to reducing the reliance on fertilizers to mitigate nutrient deficiencies in the face of global climate change.

### 2.2 Suppression of pathogens and invertebrate pests

Plant microbiomes host a range of mechanisms to combat pests and maintain plant health and resilience. Some microbes produce antimicrobial compounds which can inhibit the growth of plant pathogens (Stracquadanio et al., 2020; Kumar et al., 2021; Zehra et al., 2017; Mendoza-Mendoza et al., 2024). For example, 13 *Trichoderma* species have been reported to produce 6-pentyl-α-pyrone, an antifungal compound which can control an array of plant pathogens (Mendoza-Mendoza et al., 2024). Beneficial microbes can induce systemic resistance in plants, activating defense mechanisms that enhance plant resilience against pests (Moran-Diez et al., 2021; Kulkarni et al., 2024). The inheritance effect of plant mitogen-activated protein kinases (MAPK) primed by *Trichoderma* spp. enables next-generation seedlings to better withstand stresses (Moran-Diez et al., 2021). Competition for resources is another mechanism whereby microbes outcompete pathogenic organisms for nutrients and space around plant roots (Chepsergon and Moleleki, 2023). For example, the incidence of the pathogen *Ralstonia solanacearum* was reduced by 47% when eggplant was inoculated with rhizosphere microbiomes (Jiang et al., 2022). In the rhizosphere, *Bacillus* spp. and *Pseudomonas* spp. colonized plant roots faster than pathogenic *Fusarium* spp. and *Pythium* spp., which reduced disease pressure (Chen et al., 2024).

Microbes can also control invertebrate pests (Santoyo, 2022; Chen et al., 2023; Shi et al., 2021). Entomopathogens such as *Beauveria*, *Bacillus*, and *Metarhizium* spp. are exploited from nature to be used as biopesticides to control an array of insect pests (Glare et al., 2012). For instance, a combination of *Beauveria* strains was found to increase efficacy for control of diamondback moth (Soth et al., 2022a). The production of secondary metabolites with anti-insect properties is a common trait. These metabolites deter or inhibit pest feeding and/or reproduction (Soth et al., 2022b; Mascarin and Jaronski, 2016). For example, insect-resistant sugarcane had a higher microbiome diversity in stems than in soil, with fungi (22%) dominating over bacteria (10%), while microbiomes of susceptible sugarcane and the surrounding soil shifted toward those of resistant plants after insect damage (Li et al., 2023). This reveals a protection function of microbiomes against sugarcane insects. In other systems, nematophagous Ascomycota and Basidiomycota fungi can control pathogenic nematodes directly via parasitism and indirectly through secondary toxic metabolites (Al-Ani et al., 2022). Microbes can also modulate plant production of volatiles, influencing the emission of compounds that attract natural enemies of pests, such as predatory insects (Francis et al., 2020) and parasitoids (Contreras-Cornejo et al., 2018). Moreover, endophytic *Epichloë* fungi in grasses, which are vertically transmitted through seeds, can protect grasses against various root-feeding invertebrate pests. This protection generates approximately US$200 million annually to the New Zealand economy (Johnson et al., 2013).

Microbes’ establishment of biofilms on plant surfaces indirectly creates a physical barrier that hinders pest attachment and colonization (Velmourougane et al., 2017). Nutrient cycling and improving plant health are facilitated by beneficial microbes, making plants less susceptible to pest attacks (Iqbal et al., 2023). Furthermore, microbes trigger plant’s defense mechanisms, resulting in the synthesis of defensive proteins and phytochemicals that deter pests or reduce susceptibility to the attack (War et al., 2012).

### 2.3 Enhanced plant resilience to abiotic stresses

Plant microbiomes can foster adaptive resilience mechanisms to abiotic stresses like drought, salinity, and heat. In particular, the symbiosis of plant roots with diverse strains of plant growth-promoting rhizobacteria (PGPR) species is a key aspect of increasing drought tolerance by enhancing root proliferation, water and nutrient absorbance, osmotic regulation, antioxidant enzyme accumulation, photosynthetic activity, pigment synthesis, phytohormone adjustment, and upregulation of stress-related genes (Chieb and Gachomo, 2023). Microbes contribute to enhancing plant water use efficiency by influencing biosynthesis of the phytohormone ethylene (Suganuma et al., 1995). Ethylene regulates stomatal conductance and decreases transpiration rates, allowing the plant to better cope with drought conditions (Khan et al., 2024). Microbes can also trigger plants to generate production of osmoprotectants and antioxidants, thereby ensuring the integrity of plant cells in stress situations by regulating phytohormonal pathways and building root systems to absorb the available water (Carter et al., 2023). Like induced systemic resistance (ISR) to biotic diseases, microbes facilitate induced systemic tolerance (IST) by the establishment of resistance mechanisms against abiotic stresses (Yu et al., 2022). Microbiomes also mediate plant-microbe signaling, and they induce the upregulation of stress-responsive genes such as pathogenesis-related (PR) and pattern-recognition receptor (PRR) genes in plants (Liu et al., 2020).

Plant microorganisms also indirectly assist in the alleviation of abiotic stress. This can occur, for example, via promotion of the formation and expansion of the root system which provides a greater ability to absorb water and nutrients from the soil (Liu et al., 2021). Similarly, improved soil structure as a result of microbial activity can also conserve water and reduce the impact of drought on plant growth (Bogati and Walczak, 2022). Plant metabolites, including phytohormones and other secondary metabolites (coumarins, terpenoids, and benzoxazinoids) can stimulate microbial communities to help plants alleviate stress by improving nutrient cycling, suppressing biotic stresses, detoxifying phytotoxins, and balancing phytohormones (Su et al., 2023). The detoxification or sequestration of harmful substances by plant microbiomes (Sharma et al., 2021) also improves tolerance to stress (Montreemuk et al., 2024). Therefore, plant microbiomes help plants avoid abiotic stresses through (1) changing plant physical structure, (2) producing stress-protective substances, (3) manipulating plant resource uptake, (4) activating plant defense mechanisms, and (5) upregulating plant genes linked to response to stress.

### 2.4 Regulating phytohormones

Plant microbiomes regulate phytohormones, including auxins, cytokinins, gibberellins, abscisic acid, jasmonic acid, salicylic acid, and ethylene (Stec et al., 2016; Van der Meij et al., 2018; Ding et al., 2018; Li et al., 2019; Chen X. et al., 2019; Carvalhais et al., 2013; Reusche et al., 2013; McGuiness et al., 2019; Conway et al., 2022; Gonin et al., 2023). Auxin controls several facets of plant growth and development, such as cell elongation, maintaining apical dominance, and triggering root formation (Shani et al., 2017). Bacterial species such as *Variovorax* sp., *Pseudomonas* sp. and many other bacterial species (Gonin et al., 2023) interact with auxins in plants, with widespread impacts on growth and physiology (Conway et al., 2022; Leveau and Lindow, 2005).

Cytokinin primarily impacts plant physiological development and growth through cell division and shoot development regulation (Zurcher et al., 2013). Bacterial species like *Azospirillum* spp., *Bacillus* spp., and *Pseudomonas* spp. play a pivotal role in cytokinin signaling (Gupta et al., 2022; Hussain and Hasnain, 2011). For example, after inoculating *Platycladus orientalis* (an evergreen conifer) with *Bacillus subtilis*, Liu et al. (2013) reported a 97% and 48% increase in cytokinin contents of irrigated and drought-stressed plant shoots, respectively. As cytokinin also influences stomatal opening and closure (Hussain and Hasnain, 2011), increasing cytokinin influences plants’ resistance to biotic and abiotic stresses (Gupta et al., 2022; Todero Ritter et al., 2013). Gibberellin, another crucial plant hormone, plays a significant role in plant growth, including stem elongation, regulating seed germination, and inducing flowering (Castro-Camba et al., 2022). As with other hormones, a wide diversity of microorganisms can influence plant gibberellin levels. These include *Bacillus* spp. (Keswani et al., 2022), *Azospirillum* spp. (Cohen et al., 2009), *Aspergillus fumigatus* (Khan et al., 2011), *Penicillium* spp. (Khan et al., 2013), *Pseudomonas* spp. (Kapoor et al., 2016; Pandya and Desai, 2014), and *Rhizobium* spp. (Yanni et al., 2001). For example, inoculating gibberellin-deficient melon with *Bacillus* sp. LKE15 increased shoot length (by 33%), root length (by 9%), and plant fresh weight (by 65%) compared to the control plants (Kang et al., 2015).

Abscisic acid (ABA) has a distinct role in how plants react to environmental stress responses such as drought and salinity (Tuteja, 2007). It promotes dormancy, regulates stomatal closure, and metabolic regulation. When *Phaseolus vulgaris* roots were colonized by an entomopathogenic fungus (*Metarhizium robertsii*) and a plant pathogen (*Fusarium solani*) Hu and Bidochka (2021) reported that the plants downregulated and upregulated ABA, respectively, demonstrating that these fungi can influence *in planta* ABA. In rice and tomato, *Rhodococcus* sp. and *Novosphingobium* sp. upregulated ABA which resulted in increased plant biomass and biosynthesis (Belimov et al., 2014). Similarly, *Bacillus licheniformis* regulated ABA content, enabling *Chrysanthemum* to better withstand saline and alkaline conditions (Zhou et al., 2017).

Jasmonic acid (JA) is a phytohormone involved in various physiological processes in plants, especially stress response, defense against herbivores, and regulation of growth and development (Hewedy et al., 2023; Kulkarni et al., 2024). JA also plays another significant role in facilitating the interaction between the plant and its microbiomes (Pieterse et al., 2009). For instance, JA can stimulate beneficial bacterial and archaeal communities, including *Streptomyces*, *Bacillus*, and *Lysinibacillus* (Carvalhais et al., 2015). Similarly, salicylic acid (SA) is a key signaling molecule in plants, playing a central role in the regulation of many physiological processes, particularly in response to abiotic (Yang et al., 2023) and biotic stress (Yang et al., 2015). SA also stimulates beneficial microbes, including *Rhodanobacter*, *Sphingomonas*, and *Micromonospora*, which improved tolerance of watermelon to a *Fusarium* wilt disease (Hou et al., 2022).

Ethylene is another phytohormone that serves as a key regulator of plant growth, development, and stress responses, influencing processes like fruit ripening, leaf senescence, and abscission, while also interacting with other hormones to orchestrate adaptive plant responses (Iqbal et al., 2017; Khan et al., 2024). Besides plant genotype, microbiomes play a significant role in regulating ethylene levels in plants (Ravanbakhsh et al., 2018). Microbes such as *Azospirillum* spp*., Rhizobium* spp*., Agrobacterium* spp*., Achromobacter* spp*., Burkholderia* spp*., Ralstonia* spp*., Pseudomonas* spp*., Enterobacter* spp., and *Kluyvera ascorbata* are key species known to regulate ethylene (Blaha et al., 2006). Thus, microbiomes produce ethylene that can affect food quality and storage, as well as shelf life of ornamental flowers.

### 2.5 Facilitating food processing and preservation

Microbiomes are important for food processing and preservation, contributing to the development of various fermented and other food products, and influencing the shelf life of many more. Every day, people consume food and drinks produced through the activities of microbiomes (Siddiqui et al., 2023). The current global market for fermented food alone is valued at around USD 30 billion (Voidarou et al., 2020). For thousands of years, people have been using microbial communities for processing food, including adding distinct flavors, prolonging preservation, and improving nutritional values. Examples include cheese, soy sauce, pickled vegetables and fruit, sourdough and other breads, tempeh, yogurt, beer, cider, kombucha, and wine (Louw et al., 2023; Tamang et al., 2020; Figure 2). The following are examples of traditional foods and drinks from different cultures which use microbial processes: Japan (miso, soy sauce, and sake) (Murooka and Yamshita, 2008), South Korea (kimchi and makgeolli) (Shin et al., 2016), China (doubanjiang and baijiu) (Guan et al., 2020; Niu et al., 2023), India (dosa and idli) (Tamang, 2022), Germany (sauerkraut and beer) (Ngcamu, 2023), France (Camembert cheese and wine) (Boisard, 2003; Phillips, 2020), Italy (parmesan cheese and balsamic vinegar) (Dalby, 2009; Giudici et al., 2015), Ethiopia (injera) (Neela and Fanta, 2020), Nigeria (ogi and burukutu) (Uzogara et al., 1990), Mexico (tequila, mezcal, tepache and pulque) (Ojeda-Linares et al., 2021; Becerra-Lucio et al., 2022; Aldrete-Tapia et al., 2020), Brazil (cachaça and caiçuma) (Lima et al., 2022), Polynesia (poi) (Brown et al., 2016), Turkey (ayran and boza) (S̨anlibaba and Tezel, 2023), Iran (doogh and traditional pickles) (Meybodi et al., 2016), and Cambodia (prohok) (LeGrand et al., 2020). In food processing, certain microbes such as lactic acid bacteria (the genus *Lactobacillus*) (Giraffa et al., 2010) and fungi (*Rhizopus*, *Monascus*, and *Penicillium* spp.) (Nout and Aidoo, 2011) are intentionally introduced to transform raw ingredients into flavorful and nutritious products. During fermentation, microbes transform complex carbohydrates, proteins, and fats into new compounds, enhancing the final product’s taste, texture, and nutritional profile (Hutkins, 2008). Additionally, organic acids and antimicrobial compounds produced by these microbes help inhibit the growth of spoilage microbes, contributing to the preservation of processed foods (Sharma et al., 2020) and preventing the growth of harmful bacteria and fungi that can lead foodborne illnesses and waste of food. Techniques such as pickling, canning, and salting rely on the ability of microbes to create an environment hostile to spoilage microbes (Amit et al., 2017). For example, beneficial microbes like *Lactobacillus* bacteria create an acidic environment in fermented pickles that preserves the vegetables and enhances their flavor (Behera et al., 2020).

For future food, algae (*Spirulina* spp., *Chlorella* spp., and *Scenedesmus* spp.), bacteria (*Cellulomonas* spp. and *Alcaligenes* spp.), fungi (*Aspergillus* spp., *Penicillium* spp., and *Trichoderma* spp.), and yeast (*Saccharomyces* spp. and *Pichia* sp.) are all potential sources of protein, fiber (cellulose and poly/oligosaccharides), and vitamins (A, B, C, D, and E) (Ravindra, 2000; Graham and Ledesma-Amaro, 2023). Around 470 microorganisms were found in plant-based protein alternates, of which nearly 95% are bacteria (Roch et al., 2024). Therefore, the utilization of microbes in the food industry not only adds diversity and richness to our culinary experiences but also may provide an alternate source of future food.

## 3 Microbiomes in environmental remediation

**FIGURE 3:**
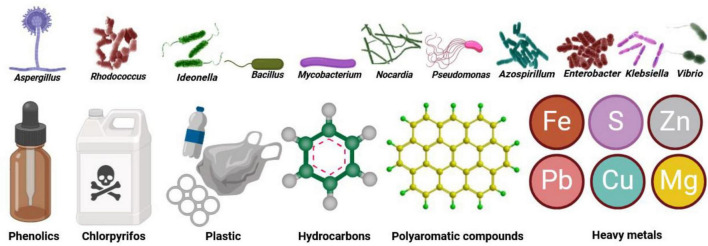
Environmental microbiomes such as *Aspergillus, Bacillus*, *Ideonella, Mycobacterium*, *Nocardia*, *Pseudomonas*, and *Rhodococcus* can break down chemicals and hydrocarbons as well as pesticidal pollutants. Other microbiomes, including *Azospirillum*, *Enterobacter*, *Klebsiella*, *Pseudomonas*, and *Vibrio* are responsible for breaking down heavy metals that are environmental pollutants.

Microbiomes have a key role in the restoration and remediation of contaminated environments (Maqsood et al., 2023; Rafeeq et al., 2023). This bioremediation, which utilizes the metabolic abilities of microbiomes to enhance the degradation of pollutants, can reduce their harmful impacts on the environment (Ayilara and Babalola, 2023). For example, *Rhodococcus rhodochrous* (bacteria) and *Aspergillus fumigatus* (fungus) have the capacity for biomethylation, a process to detoxify heavy metals and metalloids (Hartmann and Six, 2023). Additionally, the bacterium *Ideonella sakaiensis* can break down plastic wastes (Yoshida et al., 2016). Further examples include bacteria in the genera *Bacillus*, *Mycobacterium*, *Pseudomonas*, and *Nocardia* that can degrade pesticides in water and soil (Bala et al., 2022; Figure 3).

Microbiomes are essential in the process of phytoremediation whereby plants (and their microbial symbionts) filter, immobilize or transform pollutants (Sessitsch et al., 2023; Wang et al., 2024; Nathan and Ammini, 2019; Shahid et al., 2020). A microorganism able to degrade pollutants in the soil is attracted to root exudates secreted from plants. Examples of plant-microbiome heavy metal remediation systems include *Bacillus* sp. in *Arundo donax* (a perennial cane) (Sarathambal et al., 2017), *Enterobacter* and *Klebsiella* sp. in *Brassica napus* (Jing et al., 2014), *Pseudomonas* in the fern *Pteris vittate* (Manzoor et al., 2019), and *Phyllobacterium myrsinacearum* in the succulent *Sedum plumbizincicola* (Ma et al., 2013). Plants allocated up to 22% of their carbon source to their symbiont ectomycorrhizal fungi to improve nutrients uptake under nutrient inefficiency conditions (Hobbie, 2006). Overall, the synergism between microbiomes and plants improves the ability to eradicate toxins from the environment, making it an effective and sustainable environmental restoration method (Aislabie and Deslippe, 2013). Hence, using microorganisms as the driving force in establishing environmental remediation, including phytoremediation, is not only low cost and sustainable, but also is in line with the biodiversity balance and preservation.

Microbiomes have been widely used in industrial wastewater treatment. For instance, microbial communities can decompose organic matter (Fenchel et al., 2012) and remove waterborne pollutants (e.g., nitrogen fixation by bacteria such as *Enterobacter* spp., *Azospirillum* spp., *Pseudomonas* spp., *Klebsiella* spp., and *Vibrio* spp.) (Haque et al., 2020; Lamers et al., 2012). This environmentally friendly method harnesses the natural abilities of microorganisms to degrade and detoxify pollutants present in industrial effluents (Nagda et al., 2022). In combating pollutants, various strains of bacteria and fungi have been used to target specific contaminants, facilitating the breakdown of organic compounds and removing heavy metals. For instance, *Aspergillus tubingensis* isolated from the mangrove rhizosphere can absorb more than 90% of heavy metals (98% of lead, 96% of nickel, 94% of zinc, and 92% of copper) (Mahanty et al., 2020). Additionally, when using green microalgae *Chlorella vulgaris* as a co-precipitate with other heavy metals, there was a 91% and 85% reduction of phosphoric and ammonium ions, respectively (Govarthanan et al., 2020). Another study showed the bioremediation ability of the mesophilic bacteria *Lysinibacillus* sp. in reducing nickel (by 70%), chromium (by 58%), and reactive black 5 dye (by 82%) (San Keskin et al., 2018). Overall, these examples show the potential for bioremediation to offer an effective solution for removing industrial pollutants from wastewater systems.

## 4 Microbiomes in human health and medicine

### 4.1 Gut microbiomes and human health

**FIGURE 4:**
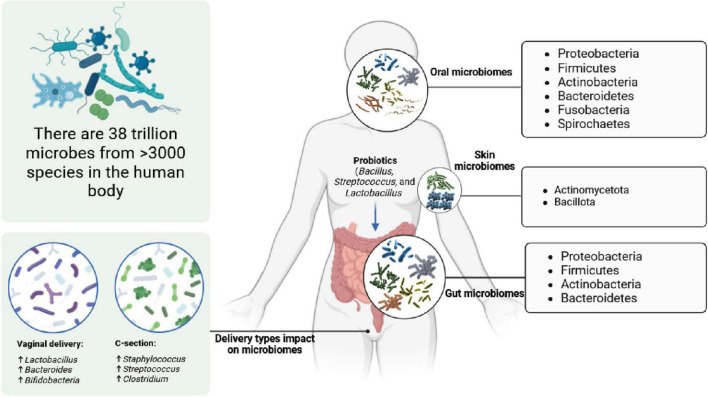
Human microbiome compositions in oral, skin, gut, and vaginal systems, and the impact of delivery method on a newborn baby’s microbiome community.

It has recently been shown that the human gut microbiome is not simply important for digestive health but plays a key role for the broader well being, physiology, inflammation, immunity, and disease status of people (Hou et al., 2022; Sender et al., 2016; Zhao et al., 2023; Li et al., 2023). The gut microbiome consists of approximately 38 trillion microbes from 3,000 species, including bacteria, viruses, and fungi. The main microbial groups of the human body are presented in Figure 4.

The gut microbiome is connected to the broader physiological state, affecting overall human health and wellbeing. For example, the gut-brain axis provides at least three well-characterized pathways of connection between the gut, brain, and broader body. As such, changes in the diversity, balance, or function of microbiomes in the gut can have profound effects on overall physical and mental health (Clapp et al., 2017; Rea et al., 2016; Campbell et al., 2023). For instance, adjusting microbial communities in the mice gut enhanced host resilience to chronic social defeat stress (Wang et al., 2020). Additionally, the human gut microbiome is known to have some roles in disease prevention, as disturbances in its composition are related to various health consequences, including autoimmune disorders, allergies, and metabolic diseases (Vijay and Valdes, 2022; Keswani et al., 2022).

Myriad foods which contain various nutrients can favorably influence the development of specific microorganisms in the gut; therefore, food choice affects microbial compositions. For instance, the intake of dietary fiber from plant-based products promotes the development of bacteria that promote digestive health and produce short-chain fatty acids, which play a role in gut health (Sun et al., 2022). However, changes in the gut microbiome related to adverse health outcomes can also be caused by food that has undergone processing and added sugar (Keswani et al., 2022). Therefore, a healthy microbiome in the gut is increasingly gaining importance as part of a holistic maintenance of health, immunity, and disease prevention through a good diet and a healthy lifestyle.

### 4.2 Microbiomes in therapeutics

The possibility to employ microbiome-targeted therapies comprising probiotics and prebiotics is an exciting approach to promotion of health and treatment of numerous diseases (Yadav and Chauhan, 2022; Ji et al., 2023). Probiotics, which are live beneficial microorganisms, when used in adequate amounts, can positively influence the amount and functions of the gut microbiome (Hemarajata and Versalovic, 2013; Das et al., 2022). These microorganisms are involved in regaining a normal microbiota balance and improving immunological fitness. They may also be used for the prevention or treatment of conditions such as inflammatory bowel disease and even allergies (Yuan et al., 2023; Cristofori et al., 2021; Simon et al., 2021). For example, a mixture of probiotics (Medilac^®^ containing *Bacillus subtilis* and *Streptococcus faecium*) in combination with Mosasal^®^ containing 5 mg of mosapride (gastroprokinetic agent) was found to reduce abdominal pain within a month (Choi et al., 2015). Unlike prebiotics, pro- and post-biotics work by stimulating the growth of healthy bacteria and reducing the number of pathogens (Davani-Davari et al., 2019; Bedu-Ferrari et al., 2022). Synbiotics, the synergistic use of probiotics and prebiotics, can also be utilized to strengthen the effects of both probiotics and prebiotics (Simon et al., 2021; Kolida and Gibson, 2011). For example, a prophylactic treatment utilizing *Lactobacillus plantarum* decreased sepsis to an overwhelming extent in newborn babies (Panigrahi et al., 2017). Thus, microbiome-based therapeutics serve as the basis for personalized medicine approaches, creating new ways of staying healthy and fighting different diseases.

### 4.3 Skin, oral, and vaginal microbiomes

Emerging research on skin, oral, and vaginal microbiomes is shedding light on their significant implications for personalized medicine and disease management. These microbial communities play crucial roles in maintaining the health and function of their respective environments (O’Mahony and Comizzoli, 2023; Prescott et al., 2017). The skin microbiome (*Propionibacterium acnes*, *Staphylococcus epidermidis*, and *Staphylococcus aureus*) (Byrd et al., 2018), for instance, influences immune responses and skin conditions (Smythe and Wilkinson, 2023), while the oral microbiome (*Firmicutes, Actinobacteria, Proteobacteria, Fusobacteriota, Bacteroidetes*, and *Spirochetes*) (Prasad et al., 2018) is linked to oral health and systemic well being (Sedghi et al., 2021). Similarly, the vaginal microbiome (*Lactobacillus* spp. and *Gardnerella vaginalis*) plays a pivotal role in reproductive health (Chen et al., 2021). For example, vaginally born babies have a beneficial microbiome (higher *Bacteroides*, *Bifidobacterium*, and *Lactobacillus*), enhancing their immune systems. In contrast, Cesarean-born babies exhibit a less diverse microbiome (mostly *Staphylococcus*, *Streptococcus*, and *Clostridium*), resembling hospital settings, potentially impacting their immune development (Coelho et al., 2021; Korpela, 2021). Examining the complicated surroundings of these microbiomes gives a new path for personalized medicine, as the variations in microbial composition can determine the level of an individual’s susceptibility to specific diseases and affect treatment outcomes (Behrouzi et al., 2019; Cullen et al., 2020). Therefore, understanding the microbiome may make it possible to develop interventions aimed at a particular microbial disbalance, enhancing the effectiveness of the therapy and minimizing side effects.

## 5 Microbiomes in biocatalyst processes

**FIGURE 5:**
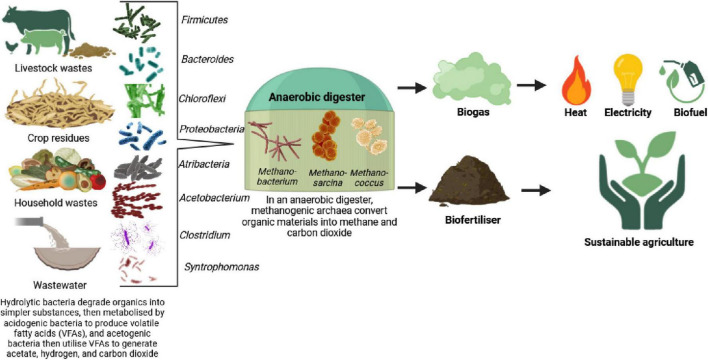
Graphical illustration of how microbiomes convert organic materials and wastewater into biogas, bioenergy, and biofertilizer.

Microbiomes have great potential to improve green processes and reduce environmental problems. Biocatalyst processes such as hydrogen production, carbon dioxide removal, and bioenergy can be significantly improved using molecular microbial engineering (Behera et al., 2022; Nagda et al., 2022). For instance, bacteria and archaea transform organic materials from animal and agricultural systems and sewage wastes into biogas. First, complex organic compounds are hydrolyzed by the bacterial phyla *Firmicutes* and *Bacteroides* into simpler substances (Mamindlapelli et al., 2021). Then, the bacterial genera *Chloroflexi, Proteobacteria*, and *Atribacteria* synthesize these substances into volatile fatty acids (VFAs). Acetogenic bacteria, including *Acetobacterium*, *Clostridium*, and *Syntrophomonas*, catabolize VFAs to form acetate, carbon dioxide, and hydrogen. Finally, the methanogenic archaeal genera *Methanobacterium*, *Methanosarcina*, and *Methanococcus* use acetate, carbon dioxide, and hydrogen as substrates for the final stage of the anaerobic process and produce methane and carbon dioxide (biogases) (Lim et al., 2020; Figure 5). Improvements are aimed at altering microbial ecosystem design to improve their efficiency, reliability, and productivity (Ramamurthy et al., 2021). Beneficial microorganisms can be introduced or environmental conditions optimized to allow microbiome-based interventions to achieve the goals of more environmentally friendly and sustainable industrial processes (Wu et al., 2021). This will allow the possibility of raising yields of recovered valuable materials such as bioproducts, biopolymers, and other value-added compounds, contributing to decreasing waste and reducing the environmental impact of industrial activities (Wiltschi et al., 2020).

Microbial biocatalysts are pivotal in advancing the sustainable production of biofuels, bioplastics, and biochemicals. Harnessing the metabolic capabilities of microorganisms allows efficient conversion of renewable resources into valuable bio-based products (Ramamurthy et al., 2021). Approximately 58 strains of bacteria, 24 species of fungi, and 17 species of yeast are known to have the ability to serve as crucial catalysts in the fermentation and transformation of biomass-derived feedstocks into ethanol, biodiesel, and other alternative fuels (Chen et al., 2014). For example, the bioethanol market is valued at USD 83.4 billion and is forecasted to increase to USD 114.7 billion by 2028 with an annual growth rate of 6.6% (MarketsandMarkets, 2024). The main microbes involved in biofuel production include *Trichoderma reesei* (which produces an enzyme that breaks down lignocellulosic biomass), *Saccharomyces cerevisiae* (utilizes hexose sugar), *Scheffersomyces stipites* (exploits pentose sugar) (Rastogi and Shrivastava, 2017), *Penicillium echinulatum* (produces cellulase and xylanase) (Todero Ritter et al., 2013), *Anoxybacillus flavithermus* (a pH and temperature stabilizer for xylanase synthesize) (Ellis and Magnuson, 2012), and *Aspergillus* spp. and *Kluyveromyces* spp. (which synthesize inulin) (Ricca et al., 2007). Combining *T. reesei*, *S. cerevisiae*, and *S. stipites* allowed a 67% yield of bioethanol from non-sterile wheat straw (Brethauer and Studer, 2014). Similarly, in the production of bioplastics, microbes contribute to the synthesis of polymers from renewable sources, offering eco-friendly alternatives to traditional petroleum-based plastics (Behera et al., 2022).

Bacterial species including *Cupriavidus necator* (Philip et al., 2007), *Pseudomonas putida* (Manoli et al., 2020), *Aeromonas hydrophila* and *Pseudomonas aeruginosa* (Chen, 2010), *Pseudomonas mendocina* (Zhang et al., 2020), *Bacillus megaterium* (Cal et al., 2021), and *Alcaligenes eutrophus* (Sathya et al., 2018), are reported to be involved in the biosynthesis of polyhydroxyalkanoates. Additionally, microbial biocatalysts are integral in the sustainable production of biochemicals, facilitating the transformation of organic materials into various high-value chemicals used in industries ranging from pharmaceuticals to agriculture (Wiltschi et al., 2020; Wu et al., 2021). Some microbes, like *Bacillus* sp., *Aneurinibacillus* sp., and *Trichoderma harzianum*, can produce lignin-degrading enzymes for sustainable processing of agro-byproducts (Sharma et al., 2022). For example, algae, bacteria, fungi, and yeasts play a significant role in transforming plant and food residues into microbial proteins, which are valuable as animal feeds (Rasool et al., 2023). This interdisciplinary approach, leveraging the capabilities of microbiomes, underscores the potential for environmentally friendly and economically viable solutions in the pursuit of a more sustainable future.

## 6 Challenges, indigenous perspectives and knowledge, and future directions

Microbiomes contribute sustainably to agriculture, human health, the environment, and biocatalyst processes. From flora and fauna sustainability to human and environmental health, microbiomes emerge as the key players in the complex regulation of ecological balance and resilience (Suman et al., 2022). Microbiomes could be pivotal in sustaining the world if their roles are correctly researched and standardized to guarantee their stability and resourcefulness in the era of climate change (Zheng et al., 2020). This illustrates the concept of a circular microbiome system, where microbiomes are interconnected across multiple dimensions of life, forming a continuous, interdependent, and virtuous cycle. The microbiome of the environment enhances soil fertility, creates a safe ecosystem, and supports sustainable food production. This, in turn, nurtures the plant’s microbiome, enabling healthy growth, resilience to diseases, and higher yields. The plant becomes food, its microbiome influencing the food microbiome, improving nutritional quality, preservation, and flavor. Once consumed, the food impacts the gut microbiome, which governs human health, digestion, and immunity. Waste generated by this process and other agricultural byproducts is transformed into bioenergy or biofuel by biocatalyst microbiomes. Finally, the final waste turns into biofertilizer, which increases plant productivity and improves microbiome diversity in the environment and soil. This interconnected process is aligned with the One Health concept, which is about interaction among humans, animals, and the ecosystem (Ma et al., 2023). Climate change has meant that around 58% of identified zoonotic diseases have emerged through pathogen spillover by altering wildlife distribution and behavior, vector and pathogen adaptability, and releasing ancient diseases from melting permafrost (Mora et al., 2022; Lim et al., 2023). As the circular microbiome system is to maintain biodiversity equilibrium and integrity, it may assist in the prevention of pathogen emergence. For example, the “Balance of Dynamic Factors” concept demonstrated the significant interactions among humans, animals, and the environment for disease prevention, health equity, and ecosystem sustainability (Zhou et al., 2024).

However, challenges need to be resolved to bring together an integrated whole-of-system approach. Addressing knowledge gaps and technical issues remains crucial to improving understanding of the microbiome and its broader applications. Attempts to understand the inner workings of microbial communities have been frustrated by their complexities, and an holistic view is required (Sergaki et al., 2018). Specifically, targeted research efforts must be carried out to close the knowledge gaps in diversity, structure, and interaction processes within microbiomes across different habitats. Moreover, technical obstacles, including developing new tools for high-throughput sequencing, bioinformatics tools, and standardized methodologies, will have to be available for use in the process (Barea, 2015). For instance, whole genome sequencing can be used to study microbiomes in different ecosystems (Aminu et al., 2024). Additionally, bioinformatic tools for data processing (FastQC, Cutadapt, DADA2, QIIME2, and MOTHUR), taxonomic profiling (Kraken2, MetaPhlAn, SILVA, and UNITE), functional profiling (HUMAnN, PICRUSt, KEGG, and EggNOG-mapper), diversity analysis (QIIME2, Phyloseq, Vegan, and MicrobiomeAnalyst), metagenomic assembly and annotation (MEGAHIT, MetaSPAdes, Prokka, and IMG/M), statistical analysis and visualization (LEfSe, ANCOM, STAMP, and ggplot2), microbial interaction and network analysis (SPIEC-EASI, CoNet, and Cytoscape), and metatranscriptomics and metabolomics (Kallisto, MetaCyc, and GNPS) can be complex and with steep learning curves for inexperienced researchers. The large amount of sequencing data generated can be computationally demanding and often requires high-performance computing infrastructure. Therefore, training and access to these tools, computing infrastructure, and dedicated bioinformatic support are all needed to help achieve the common goals.

Special consideration needs to be given to rights of Indigenous communities and peoples. As guardians of native spaces in many parts of the globe, the ecosystems they manage are often exploited (or “bio-prospected”) as a resource for microbiomes; essentially comprising biobanks of important and novel microbiomes (Warbrick et al., 2023). Māori people of Aotearoa (New Zealand), for example, practice *kaitiakitanga* (stewardship) to protect biodiversity resources, which provides insights for Western-trained researchers about holistic approaches for sustainable biological conservation (McAllister et al., 2023). For example, indigenous uses of the bark of the mānuka tree (*Leptospermum scoparium*) for its antimicrobial properties led to the discovery that mānuka honey has a wide range of health benefits (Zucchetta et al., 2022). Accompanying this is often a deep knowledge of the ecosystems themselves. This knowledge has been gained over generations of people living intimately as an integral part of these ecosystems - the first true “circular bioeconomies” - and incorporates knowledge and values of the connections between the soil and plants, the environment and communities. Recognition of their stewardship in protecting these places and the biodiversity resources they hold, which may benefit mankind, must be appropriately recognized and protected. Indeed, the interests of Indigenous communities are protected across the United Nations member countries within areas such as the Permanent Forum on Indigenous Issues, the World Intellectual Property Organization, and the Convention on Biological Diversity (Hudson et al., 2020). Article 31 of the UN Declaration on the Rights of Indigenous Peoples states that “*Indigenous people have the right to maintain, control, protect*… *manifestations of their sciences, technologies and cultures, including human and genetic resources, seeds, medicines, knowledge of the properties of fauna and flora*,…” (United Nations, 2007). This is supporting the Nagoya Protocol, a supplementary agreement to the Convention on Biological Diversity. The Nagoya Protocol includes the “*equitable sharing of benefits from using genetic resources*” (Buck and Hamilton, 2011). It is often the case that microbiome research – from the discovery of unique taxa, understanding function and ecology, through to taxonomic placement – is now supported by genetic and genomic information and, as such, underpins modern microbiology and microbiome sciences and, thereby, falls under the mandate of the Nagoya protocol.

Furthermore, Indigenous communities are often relatively impoverished and face a range of inequity issues spanning food security, access to medicine and health care, through to education, and access to financial and other support systems (Warbrick et al., 2023). Therefore, these communities may benefit significantly if a process of consultation and partnership is used, and ownership of their knowledge and microflora is appropriately protected and rewarded. There is an opportunity for science to help redress components of inequity in the systems and add value to all partners via participation, not exploitation.

Another big challenge is the establishment of solid regulatory frameworks able to control the use of microbiomes (Peixoto et al., 2022). As regulatory frameworks may vary globally, effective communication and documentation to define concepts and criteria of microbiome utilization with R&D investments to build stakeholder trust are bottlenecks to expanding the potential of microbiomes (Kostic et al., 2024). Maintaining an harmonious relationship between technology propulsion and environmental security is fundamental. Additionally, ethical considerations around this area of research become evident as the deliberate modification of microbiomes creates possibilities for unintended consequences and ecological impact (Lange et al., 2022). In the future, researchers, policymakers, and other relevant stakeholders must work together to design and develop regulatory guidelines which can be adopted as new knowledge emerges about the microbiome (Greenhough et al., 2020).

Furthermore, supporting explicit conversational spaces and active public participation, including with Indigenous communities, in developing ethical standards will be necessary to allow trustworthy practices and responsible use (Lange et al., 2022). In particular, indigenous cultural values, knowledge, and perspectives can bring and embody significant value in the life cycle of microbiome research, from discovery to development and utilization. That is, it has its own intrinsic and extrinsic values, as well as contributing to unique foods, practices, and customs of different cultures themselves. Furthermore, often, this information on the microorganisms and their relation to the environment cannot be obtained elsewhere; it sits with and is held by the people and is often passed across and down generations as oral knowledge. The Western approach of sharing knowledge in published format (print or online) cannot access this deep well of information.

Integrating different disciplines, such as microbiology, genetics, ecology, and computational sciences, is another challenge (Cullen et al., 2020). While some studies have explored a system approach to interdisciplinary and transdisciplinary collaboration (Meisner et al., 2022; Tomasulo et al., 2024), the practical aspects of such frameworks requires breaking technical and structural barriers. Sharing knowledge and cooperation are the keys in fully capturing the value of microbiome research. Moreover, retrieving multiple data sources from different methods and tools entails another challenge, thus necessitating the creation of common approaches. Since microbiome research is progressing rapidly, the future course should give precedence to the breaking down of silos and promoting collaboration of scientists from all disciplines (Greenhough et al., 2020). Tomasulo et al. (2024) proposed a model for a healthy ecosystem and encouraged innovative microbiome research via active collaboration among all relevant stakeholders to achieve this goal. Multidisciplinary research team creation, furthering cross-training, and establishing open data-sharing platforms will be key to potentially using microbiomes (Acosta et al., 2022; Amann et al., 2019; Tomasulo et al., 2024; Meisner et al., 2022). Adopting modern technologies and techniques, e.g., advanced sequencing methods and computational modeling, will lead to groundbreaking findings in basic and applied genomics (Bianconi et al., 2023). Therefore, overcoming these challenges will be instrumental in the implementation of interdisciplinary work in microbiome research, and this will mark the beginning of a new era wherein the complex interactions between microbiome function and adaptation to different ecosystems will be understood in totality.

## 7 Conclusion

The highly multifaceted nature of microbiomes plays a pivotal role in interdisciplinary ecosystems. From their critical involvement in agriculture and food production, through addressing environmental issues, impacting human health and finding novel and eco-friendly biocatalyst processes, microbiomes are a sustainable tool for a greener future. As a part of nutrient cycling, pest and disease management, and cleansing of environmental toxins, microbiomes offer a sustainable way to reduce environmental pollutants. They are also significant for human health, contributing largely to whole-body well being, immune system functioning, prevention of diseases, and personalized medicines. In biocatalyst processes, microbial community activity ranges from optimizing processes involved in wastewater treatment to fermentation of biofuels, bioplastics, and biochemicals. Such microbiome-focused innovations are important for sustainable ecological balance, biodiversity conservation, and assured eco-friendly industrial practices.

These opportunities are also accompanied by the challenges of knowledge gaps, integrating Indigenous knowledge, technical limitations, regulatory frameworks, interdisciplinary collaboration, and ethical considerations, all of which require attention. Breaking interdisciplinary barriers, creating a collaborative culture, and adopting innovative technologies will be key stepping stones in tackling these challenges. Constant assessment, ethical associations, and progressive points of view are essential for effective microbiome utilization. By achieving this, the true capabilities of microbiomes can be unveiled, allowing us to better understand microbial communities and how they interact with the ecosystem. The multifaceted benefits of microbiomes will provide both economically viable and environmentally friendly mechanisms for plant production, improve ecological equilibrium and biodiversity conservation, reduce industrial pollutants, and offer opportunities for improving human health and indigenous people’s values.

A clear roadmap to fully harness the multifaceted benefits of microbiomes is necessary. Future studies should focus on existing systems-based approaches to interdisciplinary and transdisciplinary collaboration (Meisner et al., 2022; Tomasulo et al., 2024) to address knowledge gaps, to include Indigenous knowledge, and to develop advanced tools like meta-omics and biological synthesis. Researchers and policymakers should work together to establish ethical regulatory frameworks, enrich international partnerships, and guarantee equitable access to microbiome innovation. Practical applications across academic disciplines mentioned in this review must be expanded with long-term planning and public intervention. In order to be able to add to the existing literature (Aminu et al., 2024; Buerger et al., 2023; Kostic et al., 2024), this roadmap envisages ethical awareness, Indigenous communities and practical applications, to provide greener, healthier, and more equitable sharing through microbiome innovations. Achieving this will reveal how microbiomes can be utilized as future tools for sustainable agricultural development, enhancing human health, conserving biodiversity, and harmonizing equity among researchers, policymakers, the public, and Indigenous peoples.
